# Super Mg^2+^ Conductivity around 10^–3^ S cm^–1^ Observed in a Porous Metal–Organic Framework

**DOI:** 10.1021/jacs.2c01612

**Published:** 2022-05-04

**Authors:** Yuto Yoshida, Teppei Yamada, Yuan Jing, Takashi Toyao, Ken-ichi Shimizu, Masaaki Sadakiyo

**Affiliations:** † Department of Applied Chemistry, Faculty of Science Division I, Tokyo University of Science, 1-3 Kagurazaka, Shinjuku-ku, Tokyo 162-8601, Japan; ‡ Department of Chemistry, Graduate School of Science, The University of Tokyo, 7-3-1 Hongo, Bunkyo-ku, Tokyo 113-8654, Japan; § Institute for Catalysis, Hokkaido University, N-21, W-10, Sapporo, Hokkaido 001-0021, Japan

## Abstract

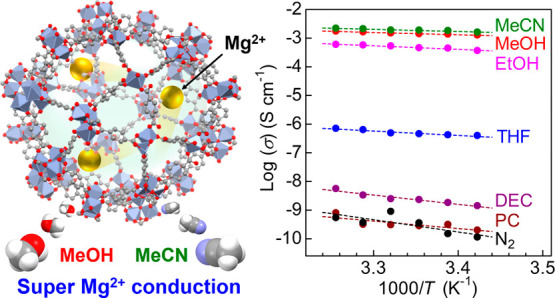

We first report a solid-state crystalline “Mg^2+^ conductor” showing a superionic conductivity of around 10^–3^ S cm^–1^ at ambient temperature, which was obtained using the pores of a metal–organic framework (MOF), MIL-101, as ion-conducting pathways. The MOF, MIL-101⊃{Mg(TFSI)_2_}_1.6_ (TFSI^–^ = bis(trifluoromethanesulfonyl)imide), containing Mg^2+^ inside its pores, showed a superionic conductivity of 1.9 × 10^–3^ S cm^–1^ at room temperature (RT) (25 °C) under the optimal guest vapor (MeCN), which is the highest value among all Mg^2+^-containing crystalline compounds. The Mg^2+^ conductivity in the MOF was estimated to be 0.8 × 10^–3^ S cm^–1^ at RT, by determining the transport number of Mg^2+^ (*t*_Mg^2+^_ = 0.41), which is the level as high as practical use for secondary battery. Measurements of adsorption isotherms, pressure dependence of ionic conductivity, and in situ Fourier transform infrared measurements revealed that the “super Mg^2+^ conductivity” is caused by the efficient migration of the Mg^2+^ carrier with the help of adsorbed guest molecules.

## Introduction

Efficient magnesium ion (Mg^2+^) conduction in a solid is an important issue for the realization of a solid-state Mg ion battery, which is expected as one of the ideal energy storage devices that do not require the use of rare elements such as Li.^1^ One of the critical problems in current solid-state Mg^2+^ conductors regarding their use for batteries is their low conductivity. Normally, the electrolyte of a secondary battery requires a practical conductivity of around 10^–3^ S cm^–1^ at operating temperatures.^2^ There are numerous reports of Li^+^ conductors showing superionic conductivity above 10^–3^ S cm^–1^ at room temperature (RT),^3,4^ while we found no report of such RT conductivity for Mg^2+^ conduction in crystalline solids. The main reason for the suppressed conductivity of Mg^2+^ in solids is the strong electrostatic interaction with neighboring anions because conventional solid-state materials tend to have closely packed crystal structures with the included Mg^2+^ carrier.^5^

Recently, metal–organic frameworks (MOFs) have been widely studied as novel ion-conductive materials with various ionic carriers such as protons (H^+^)^6,7^ and hydroxide ions,^8^ showing excellent conductivity at ambient temperatures. The porous structures of MOFs provide great opportunities for constructing efficient ion-conductive pathways inside the pores, which often consist of adsorbed guest molecules, called “conducting media” (e.g., H_2_O in H^+^ conduction). However, in the case of Mg^2+^,^9−12^ there is a lack of reports of high “Mg^2+^ conduction” inside the MOF pores, and thus, the creation of a highly Mg^2+^-conductive MOF is still a challenging issue. We recently reported on “Mg^2+^ conduction” with a conductivity of around 10^–4^ S cm^–1^ at RT inside the MOF pore, which is assisted by adsorbed guest molecules such as MeOH, introduced as anhydrous organic vapors.^13^ However, the conductivity is still insufficient for practical use.

Here, we demonstrate for the first time super Mg^2+^ conductivity of around 10^–3^ S cm^–1^ in a solid at RT using MOF pores and the adsorbed guest molecules. Previously, we reported that small organic molecules such as MeOH strongly accelerate the Mg^2+^ conductivity in the MOF (Mg-MOF-74⊃{Mg(TFSI)_2_}_*x*_) (TFSI^–^ = bis(trifluoromethanesulfonyl)imide), and that the mobile Mg^2+^ carrier would be coordinated or solvated species formed in MOF pores.^13^ This suggested the efficient migration of a coordinated Mg^2+^ carrier, whose size is much larger than the lone Mg^2+^, as it requires a relatively large pore size of the mother framework as the optimal ion-conducting pathway. In this study, we used MIL-101, which has large three-dimensional (3-D) pores (maximum approximately 32 Å),^14^ as the mother framework and prepared MIL-101⊃{Mg(TFSI)_2_}_*x*_ (MIL-101 = Cr_3_O(NO_3_)(H_2_O)_2_(bdc)_3_, where H_2_bdc is terephthalic acid, *x* = 0–1.7) that only includes Mg(TFSI)_2_ as the Mg^2+^ carrier in it. The samples showed a high ionic conductivity under anhydrous organic vapors, and MIL-101⊃{Mg(TFSI)_2_}_1.6_ exhibited superionic conductivity of 1.9 × 10^–3^ S cm^–1^ under MeCN vapor at RT, which is the highest conductivity among all Mg^2+^-containing crystalline solid-state materials. The transport number of Mg^2+^ (*t*_Mg^2+^_), that is, the direct evidence of Mg^2+^ transport, was determined to be *t*_Mg^2+^_ = 0.41, confirming an Mg^2+^ conductivity of 0.8 × 10^–3^ S cm^–1^ at RT in the MOF. The role of the adsorbed guest molecules for the “Mg^2+^ conduction” in the MOF pores was also studied.

## Experimental Section

### Synthesis of MIL-101

MIL-101 was synthesized using the solvothermal method. Cr(NO_3_)_2_·9H_2_O (15.2 g, 38.1 mmol) and terephthalic acid (H_2_bdc) (6.24 g, 37.5 mmol) were stirred in deionized water (150 mL) for several minutes at RT. The mixture was placed in a Teflon-lined autoclave and heated at 200 °C for 24 h. The precipitate was collected by centrifugation. It was washed by repetition of immersion in dimethylformamide and centrifugation. After that, it was stirred in MeOH at 70 °C for 1 d (the solvent was replaced with fresh MeOH two times during the day). The green powder was collected by centrifugation and dried at 70 °C for 24 h (yield: 10.9 g, 36%). Elemental analysis. Calcd (for Cr_3_O(NO_3_)(H_2_O)_2_(C_8_O_4_H_4_)_3_(H_2_O)_0.8_(CH_3_OH)): C, 37.12%; H, 2.69%; N, 1.88%. Found: C, 37.34%; H, 2.75%; N, 1.88%.

### Synthesis of MIL-101⊃{(MgTFSI)_2_}_*x*_ ([Cr_3_O(NO_3_) (H_2_O)_2_(bdc)_3_]⊃{(MgTFSI)_2_}_*x*_, *x* = 0, 0.5, 1.1, 1.6, and 1.7)

Mg(TFSI)_2_ was introduced into MIL-101 through slow evaporation of EtOH from a EtOH solution of Mg(TFSI)_2_. MIL-101 (400 mg, 0.495 mmol) was soaked in EtOH solution (20 mL) of [Mg(H_2_O)_6_](TFSI)_2_·2H_2_O (0, 164, 323, 486, and 600 mg are used for the samples of *x* = 0, 0.5, 1.0, 1.6, and 1.7, respectively) in a test tube. It was then heated at 85 °C for 7 d.

### Physical Measurements

X-ray powder diffraction (XRPD) measurements were performed in air using Rigaku MiniFlex600 (Cu Kα). Adsorption isotherms for N_2_ (77 K) and MeCN vapor (298 K) were measured using BELSORP-max (Microtrac BEL, Inc.). Before the measurements, the samples were dried under vacuum at 130 °C for a night. Inductively coupled plasma atomic emission spectroscopy (ICP–AES) measurements were carried out using SPECTRO ARCOS (Hitachi High-Tech Science Corporation). The sample (about 20 mg) was added with nitric acid (2 mL) and diluted to 50 mL with deionized water. The supernatant solution was used for ICP–AES measurements to determine the concentration of Mg.

### Conductivity Measurements

**Figure 1:**
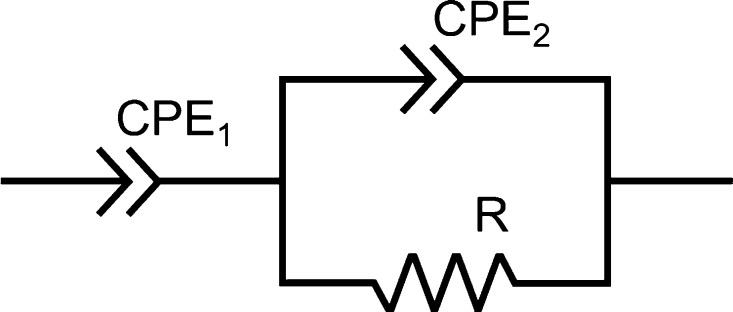
An equivalent circuit used for fitting of the impedance spectra of the sample under THF vapor.

The alternating current (ac) impedance measurements were carried out with the two-probe method using a Solartron 1260/1296A impedance analyzer for the frequency range of 1 Hz to 10 MHz. Two porous silver electrodes were attached to the compacted pellet (3 mm ϕ) in a homemade sealed cell, as previously reported.^13^ The temperature was controlled in an incubator, SU-222 (ESPEC, Inc.). The amounts of N_2_ gas flows were controlled by mass flow controllers. The samples were dried under N_2_ flow at 130 °C overnight to remove adsorbed water molecules before the measurements. After that, ionic conductivity was measured under the dry N_2_ flow or various guest vapors of MeOH, EtOH, MeCN, tetrahydrofuran (THF), diethyl carbonate (DEC), and propylene carbonate (PC), produced by bubbling each anhydrous organic solvent with the dry N_2_ in the temperature range of 19–34 °C. The resistance of the sample was estimated by a semicircle fitting (for the samples under DEC, PC, and N_2_). In the case of the sample under THF, a typical equivalent circuit, as shown in Figure 1, was applied. The resistance of the sample was estimated by fitting with the equivalent circuit. In the case that the semicircle fitting or the equivalent circuit fitting were difficult (for the samples under MeCN, MeOH, and EtOH), we estimated the sample resistance from the real part of the impedance observed on the inflection point from the sample resistance to electrode–sample resistance or *Z*′-axis intercept (for the sample under MeOH).

### Measurements of Transport Number of Mg^2+^

According to previous reports,^15,16^ the transport number of Mg^2+^ in the sample was estimated by measuring the direct current (dc) of the cell, Mg|MIL-101⊃{Mg(TFSI)_2_}_*x*_(MeCN)_*n*_|Mg. The *x* = 1.6 sample was preliminarily dried at 130 °C under vacuum for a night. The cell was constructed using a sealed cell. All experimental manipulations for the construction of the cell were performed in a glove bag filled with Ar gas with MeCN vapor. The powder of the sample was sandwiched with metal Mg foil and pressed into a pellet (5 mm ϕ) under MeCN vapor. The dc current was measured using a Vertex10A (IVIUM Technologies, Inc.) potentiostat/galvanostat by applying 0.3 V at 60 °C. Before and after the polarization, ac impedance measurements were also performed using a Solartron 1260/1296A impedance analyzer for the frequency range of 1 Hz–10 MHz to estimate the resistance between the electrode and the sample. The transport number of Mg^2+^ was calculated with the Bruce–Vincent method^17^ using the following equation
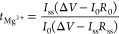
where Δ*V*, *I*_0_, *I*_ss_, *R*_0_, and *R*_ss_ correspond to the applied voltage, initial current, current at steady state, initial resistance, and resistance at steady state, respectively. We performed four experiments with the same setup and estimated the final value of *t*_Mg^2+^_ as an average of these four experiments.

### In Situ Fourier Transform Infrared Spectroscopy Measurements

In situ Fourier transform infrared (FT-IR) spectra were recorded at 30 °C using a JASCO FT/IR-4200 with an mercury–cadmium–telluride detector. A sample (40 mg) was pressed to obtain a self-supporting pellet (ϕ = 2 cm). The obtained pellet was placed in the quartz IR cell with CaF_2_ windows connected to a conventional gas flow system. Prior to the measurement, the sample pellet was heated under He flow (100 cm^3^ min^–1^) at 130 °C overnight. After cooling to 30 °C under the He flow, 2 μL of MeCN was injected to the sample. Spectra were measured accumulating 20 scans at a resolution of 4 cm^–1^. A reference spectrum taken at 30 °C under He flow was subtracted from each spectrum.

## Results and Discussion

### Preparation and Characterization

**Figure 2:**
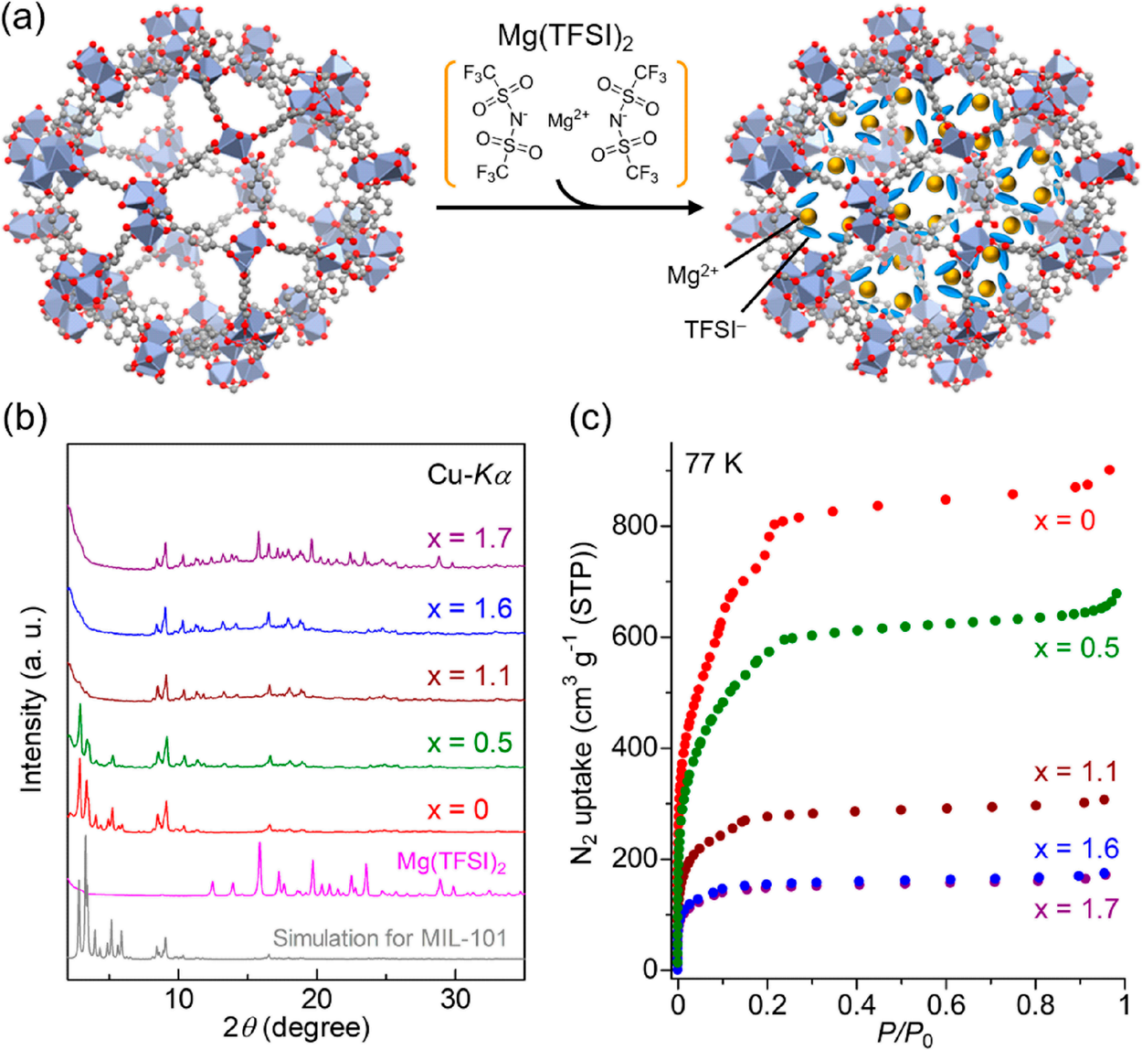
(a) Schematic illustration of Mg(TFSI)_2_ inclusion inside the pores of MIL-101. (b) XRPD patterns of the samples of *x* = 0–1.7. (c) Adsorption isotherms of the samples of *x* = 0–1.7 for N_2_ at 77 K.

The samples, MIL-101⊃{Mg(TFSI)_2_}_*x*_ (*x* = 0–1.7) (Figure 2a), were prepared by introduction of Mg(TFSI)_2_ into blank MIL-101 (Figure S1) through slow impregnation with gradual evaporation of the EtOH solvent for several days, according to our previous report.^13^ The Mg content in the sample was quantified using ICP–AES. Figure 2b shows XRPD patterns of the prepared samples with different Mg(TFSI)_2_ contents (*x* = 0–1.7). The peaks from bulk Mg(TFSI)_2_ crystals (accurately described as [Mg(H_2_O)_6_](TFSI)_2_·2H_2_O) were observed above the *x* = 1.7 sample, whereas the MIL-101 peaks were observed in all samples, indicating that MIL-101 could incorporate the Mg(TFSI)_2_ inside its pores below the molar amount of *x* = 1.6. Note that, we confirmed that the intensity of weakened peaks at low angle (below 7°) in the samples above *x* = 1.1, recovered by exclusion of the included salts through immersing the sample in the pure solvent. This is indicative that the decrease in the peak intensity at the low angle was not due to decomposition of the framework but derived from some other reasons such as change in electron density in the pores or weakening of the long-range order of the framework occurred by inclusion of the salts. The incorporation of Mg(TFSI)_2_ was confirmed by N_2_ adsorption measurements at 77 K (Figure 2c). The adsorption amount decreased with the increasing Mg(TFSI)_2_ content of the samples, with almost no difference between the *x* = 1.6 and 1.7 samples, confirming that the incorporation of Mg(TFSI)_2_ inside the pores occurred below *x* = 1.6.

### Mg^2+^ Conductivity

**Figure 3:**
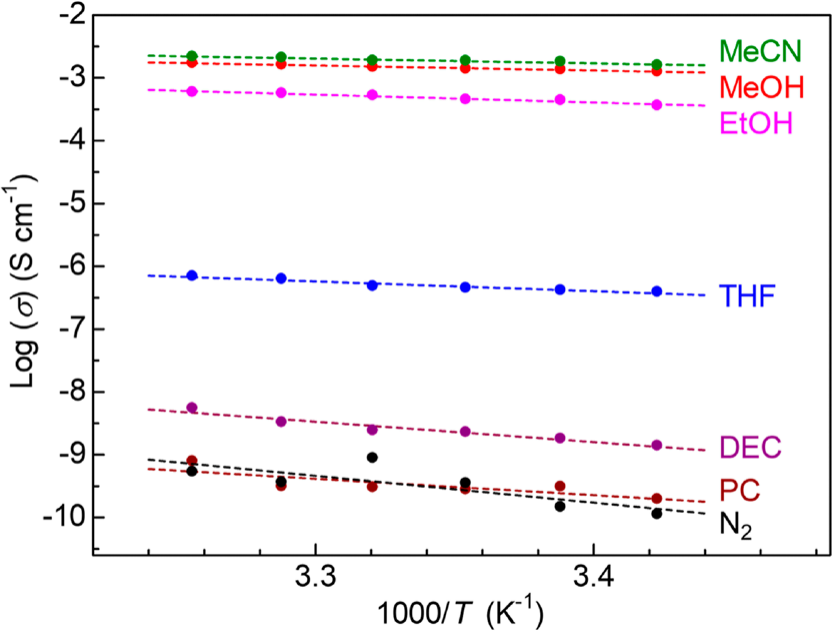
Temperature dependence of ionic conductivity of MIL-101⊃{Mg(TFSI)_2_}_1.6_ under various guest vapors or dry N_2_.

To enclose the ionic conductivity of the MOF under optimal conditions, we performed ac impedance measurements under various anhydrous guest vapors or dry N_2_ after complete dehydration of the sample. Examples of the obtained Nyquist plots are shown in Figure S2. In the case of the sample under THF vapor, a typical plot consisting of both a semicircle derived from the sample resistance and a part of increasing impedance due to the huge resistance between the blocking electrode and the sample was observed. Thus, the sample resistance could be determined by fitting with the equivalent circuit, as described above. The samples under DEC, PC, and N_2_ only showed a part of the semicircle from the sample resistance due to their high resistivity. In contrast, in the case of the samples under MeOH, EtOH, and MeCN, a part of the electrode-sample impedance or the inflection point from the sample impedance to it was clearly observed because of the low resistivity of the sample, which is similar to the case of other highly ion-conductive materials.^18^ It is clear that the drastic change in the sample resistance was observed just by changing the vapor atmosphere around the sample, as is the case with our previous report.^13^ Since we could not observe the divided components of bulk and grain boundary contributions in the Nyquist plots, it was difficult to be sure of whether the ionic conductivity (i.e., the observed sample resistance) is mainly derived from the bulk or grain boundary, only by the impedance measurements. However, several facts could confirm that the changes in the ionic conductivity correspond to the changes in the bulk conductivity. For example, as we reported,^13^ Mg(TFSI)_2_ itself does not show such a change in ionic conductivity under vapors, meaning that there is no possibility that slight amount of Mg(TFSI)_2_ located on the outside of the pores make the ion-conducting pathway in the grain boundary region. In addition, the clear relationship between guest adsorption and ionic conductivity of the Mg-included sample, as described below, cannot be explained by the hypothesis that the ionic conductivity of the sample is derived from the grain boundary. Therefore, we could attribute the observed conductivity of the sample to the bulk conductivity. The temperature dependence of ionic conductivity of MIL-101⊃{Mg(TFSI)_2_}_1.6_, in which the maximum amount of Mg^2+^ was incorporated, is shown in Figure 3. The sample showed a strong dependence of the ionic conductivity on guest vapors, that is, vapor-induced superionic conduction, which is similar to our previous report.^13^ Surprisingly, MIL-101⊃{Mg(TFSI)_2_}_1.6_ showed superionic conductivity (1.9 × 10^–3^ S cm^–1^) at RT under MeCN vapor, which was the highest value among all Mg^2+^-containing crystalline compounds (Table S1). Very high conductivity values were also observed at RT under other small guest molecules such as MeOH (1.4 × 10^–3^ S cm^–1^) and EtOH (4.5 × 10^–4^ S cm^–1^), and moderate conductivity was observed at RT under THF (4.5 × 10^–7^ S cm^–1^). In contrast, almost no enhancement in the ionic conductivity was observed under DEC or PC vapor, and thus, the sample presented an insulating character (<10^–8^ S cm^–1^), which was similar to the dry N_2_ condition. Note that the dc conductivity of the samples (*x* = 0 and 1.6) was not observable (at least below 10^–8^ S cm^–1^), confirming that the effect of electron conduction of the MOF is negligible and that the conductivity observed in the ac impedance measurements is derived from the ionic conductivity. The samples of *x* = 0, 0.5, 1.1, and 1.6 showed ionic conductivity under MeCN (Figure S3). The conductivity increased almost monotonically with increasing Mg(TFSI)_2_ content in the pores, indicating that the ionic conductivity of the *x* = 1.6 sample was derived from the included Mg(TFSI)_2_.

**Figure 4:**
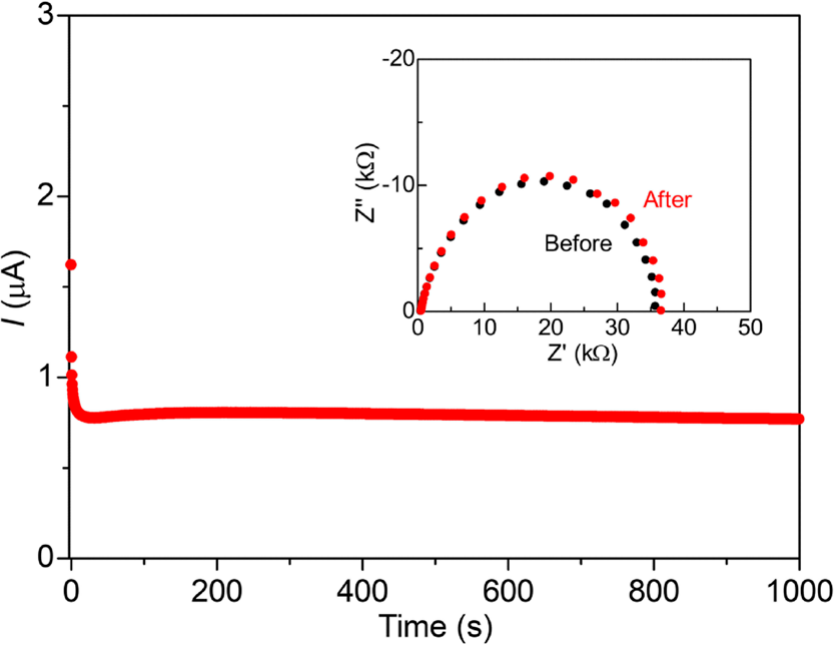
dc polarization curve of Mg|MIL-101⊃{Mg(TFSI)_2_}_1.6_(MeCN)_*n*_|Mg with 0.3 V at 60 °C, exemplified by the case with *t*_Mg^2+^_ = 0.44 (other experiments gave *t*_Mg^2+^_ = 0.37, 0.46, and 0.36 with the same setup). The inset shows the Nyquist plots of the cell before and after the polarization.

Because the sample included both Mg^2+^ and TFSI^–^ in the pores, conducting ionic species should be identified to state the “Mg^2+^ conduction” in the MOF. To quantify the contribution of Mg^2+^ conduction to the ionic conductivity, we evaluated the transport number of Mg^2+^ (*t*_Mg^2+^_) through dc polarization measurements with nonblocking electrodes, as we previously reported.^13^ The value for *t*_Mg^2+^_ under MeCN vapor for the *x* = 1.6 sample was estimated to be 0.41 (the average of four experiments) (Figure 4), which directly indicated that almost half the ionic conductivity in the MOF was truly derived from “Mg^2+^ conduction,” and that MIL-101⊃{Mg(TFSI)_2_}_1.6_ showed “super Mg^2+^ conductivity” (σ_Mg^2+^_ = 0.8 × 10^–3^ S cm^–1^), at RT under MeCN. This is the first crystalline solid showing a practical Mg^2+^ conductivity of around 10^–3^ S cm^–1^ at ambient temperature. The activation energy of the ionic conduction in the *x* = 1.6 sample under MeCN was estimated to be 0.18 eV, much lower than that of reported Mg^2+^-containing compounds (0.8–1.6 eV)^5,19−23^ and similar to MgSc_2_Se_4_ (0.2 eV),^24^ which is one of the best crystalline inorganic Mg^2+^ conductors, suggesting that efficient ion-conducting pathways were formed inside the MOF pores.

### Relationship between Ionic Conductivity and Guest Adsorption

**Figure 5:**
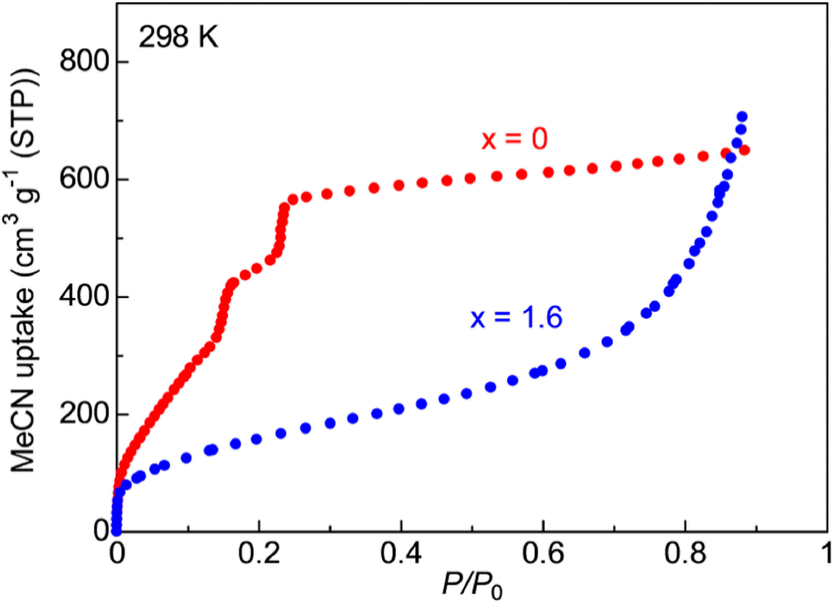
Adsorption isotherms of the samples of *x* = 0 and 1.6 for MeCN vapor at 298 K.

To clarify the role of the guest molecules for the super Mg^2+^ conduction inside the MOF, we measured adsorption isotherms for MeCN vapor (Figure 5). The pristine MIL-101 (*x* = 0) showed a large amount of MeCN adsorption at the low-pressure region, confirming its large pore volume and high affinity for MeCN. When the Mg(TFSI)_2_ was incorporated into the MOF, the adsorption of MIL-101⊃{Mg(TFSI)_2_}_1.6_ at the low-pressure region (∼0.2 *P*/*P*_0_) was suppressed, and the shape of the isotherms was remarkably changed compared with *x* = 0. This change in adsorption isotherms was indicative that the incorporation of Mg(TFSI)_2_ does not only lead to a decrease in pore volume but changes the affinity of the remaining pores for guest molecules, that is, causes changes in the host–guest interaction. Because the *x* = 1.6 sample presented type II-like isotherms, different types of adsorption sites or adsorption processes corresponding to the low- and high-pressure regions should exist. In the low-pressure region, the adsorption might be due to strong interactions, such as chemisorption by Mg^2+^, with coordination bonds or strong binding by the framework. Above this region, the adsorption might be due to weaker interactions such as the guest–guest interaction with intermolecular forces. The adsorption in the *x* = 1.6 sample at the low-pressure region reached ∼10 MeCN molecules (9.5 at 0.1 *P*/*P*_0_) per formula (Figure S4), which is near the 9.6 that is in good agreement with the amount to form hexa-coordinated Mg^2+^ species by chemisorption (i.e., [Mg(MeCN)_6_]^2+^)^25^ in MIL-101⊃{Mg(TFSI)_2_}_1.6_. This suggests that the superionic conduction occurs under the presence of both the coordinated or solvated Mg^2+^ species and additional uncoordinated MeCN molecules adsorbed in the higher-pressure region.

**Figure 6:**
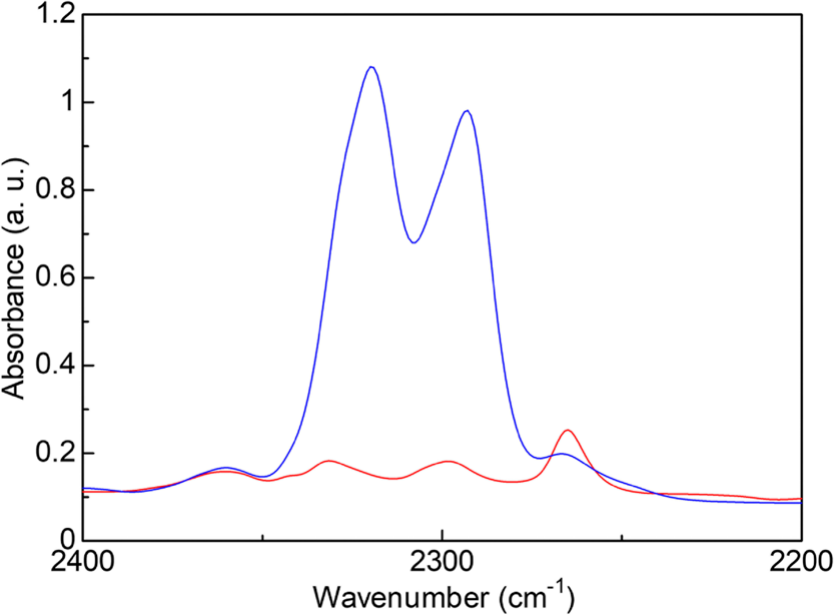
FT-IR spectra of adsorbed MeCN species on the samples of *x* = 0 (red) and 1.6 (blue) at 30 °C (*t* = 60 min). At *t* = 0 min, 2 μL of MeCN was introduced to the FT-IR cell containing a preheated sample.

To get the direct evidence of the presence of the coordinated Mg^2+^ carries in the MOF, we performed in situ FT-IR measurements for *x* = 0 and 1.6 under the MeCN vapor after complete dehydration of the samples. As shown in Figure 6, there is a clear difference between the absorption spectra of *x* = 0 and 1.6 in the region of 2250–2350 cm^–1^, which is attributable to the absorption by MeCN. According to the literature, a MeCN molecule gives two characteristic absorption bands, namely, ν_2_ (CN stretching) and ν_3_ + ν_4_ (combination of CH_3_ bending and CC stretching), in this region.^26^ In pure MeCN liquid (i.e., uncoordinated MeCN molecule), the ν_2_ and ν_3_ + ν_4_ bands are observed at around 2253 and 2293 cm^–1^, respectively, while the MeCN coordinating to Mg^2+^ gives the shifted bands at around 2287 (for ν_2_) and 2315 cm^–1^ (for ν_3_ + ν_4_).^26^ In the case of the Mg^2+^-included MOF (*x* = 1.6), these two different states of MeCN were clearly observed. The main strong peaks at 2293 and 2320 cm^–1^ were attributable to the MeCN coordinating to Mg^2+^, which clearly confirms that the coordinated species, such as [Mg(MeCN)_6_]^2+^, were formed in the *x* = 1.6 under the MeCN vapor. In contrast, the spectra of the pristine MOF (*x* = 0) was mainly composed of the free MeCN [observed at 2265 cm^–1^ (for ν_2_)], while the shifted bands were slightly observed, which might be derived from the MeCN coordinating to some defects or open metal sites on the MIL-101 framework. This result is consistent with our previously reported hypothesis that the migration of the coordinated or solvated species occurs in the Mg^2+^-included MOF under guest vapors.^13^ According to these results, we think that the excellent ionic conductivity with the remarkably low activation energy of the *x* = 1.6 under the guest was caused due to the decreased electrostatic interaction (i.e., elongated distance) between the carrier and some of trapping sites and the lowered friction between the migrating carrier and framework or surrounded guest molecules.

**Figure 7:**
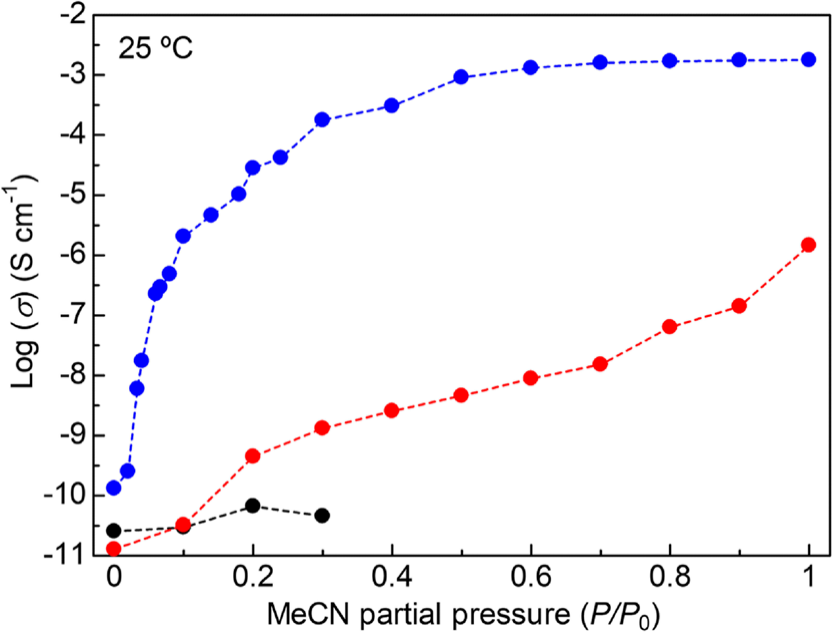
Dependence of ionic conductivity on MeCN partial pressure. Blue, red, and black colors correspond to the samples of *x* = 1.6, *x* = 0, and bulk Mg(TFSI)_2_, respectively.

To obtain a more precise picture of the role of adsorbed MeCN molecules for the ionic conduction, we measured the dependence of ionic conductivity under the varied partial pressure of MeCN vapor in the range from 0 to 1 *P*/*P*_0_ at RT, using a homemade conductivity evaluation system including multiple gas flow lines (Figure S5). The ionic conductivity of the *x* = 1.6 sample drastically increased with increasing MeCN partial pressure and finally reached above 10^–3^ S cm^–1^ (Figure 7). The conductivity drastically increased in the low-pressure region, particularly below 0.3 *P*/*P*_0_, and saturated at ca. 0.5 *P*/*P*_0_. This clearly indicated that the adsorption processes or adsorption sites, which depend on the partial pressure, are some of the critical factors for ionic conduction in the MOF. It is to note that the trend of ionic conductivity is different from that of bulk Mg(TFSI)_2_ salt, which shows the negligible change in conductivity below 0.3 *P*/*P*_0_, and above 0.4 *P*/*P*_0_, it was impossible to measure the conductivity due to deliquesces. The different vapor response in ionic conductivity shows that the ionic conduction in the *x* = 1.6 sample was not derived from bulk Mg(TFSI)_2_ on the surface of the MOF as a possible impurity, as mentioned above. It is also important to note that the pristine MIL-101 (*x* = 0) did not show such high conductivity at any partial pressure. These results demonstrated that the superionic conduction in the *x* = 1.6 sample was indeed derived from the Mg(TFSI)_2_ salt incorporated into the MOF pores and the adsorbed MeCN molecules.

**Figure 8:**
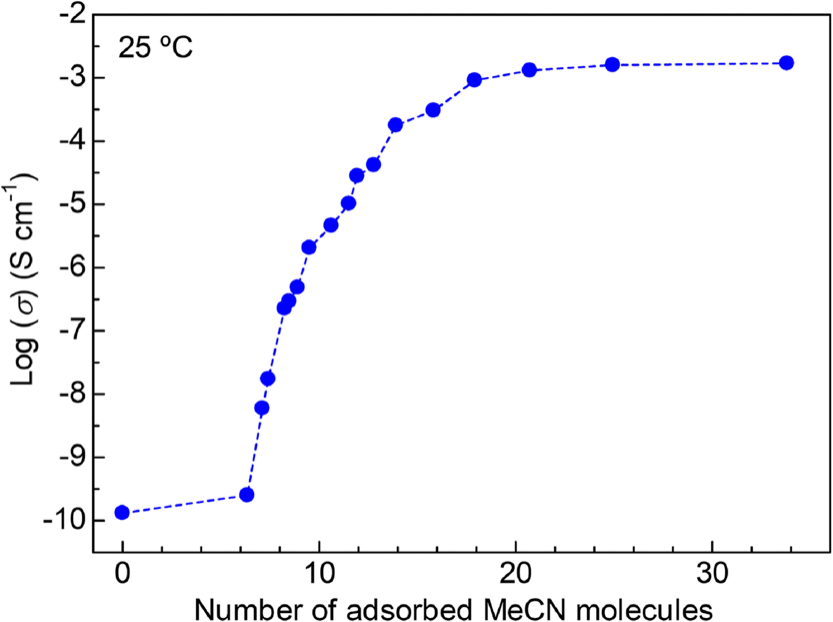
Relationship between ionic conductivity and adsorbed MeCN molecules per formula of the *x* = 1.6 sample.

From the combination of the results of adsorption isotherms and the partial pressure dependence of ionic conductivity, we could clarify the relationship between the ionic conductivity and the number of adsorbed MeCN molecules (Figure 8). Notably, the ionic conductivity of the *x* = 1.6 sample did not show the monotonic increase with the number of adsorbed MeCN molecules but showed a drastic rise within a specific amount (approximately from 7 to 16) of MeCN molecules per formula, which corresponds to the region near the formation of coordinated species of Mg^2+^ (9.6 MeCN molecules). This clearly indicated that the formation of coordinated Mg^2+^ and some additional MeCN molecules adsorbed in the pores critically contribute to the “super Mg^2+^ conduction” in the MOF. It was also revealed that additional adsorption above 18 MeCN molecules, which corresponds to the MeCN molecules bound by a weak interaction such as the guest–guest interaction, does not strongly contribute to the enhancement of the ionic conductivity. The detailed studies on the dynamics of the superionic conduction in the MOF are still undergoing, while we believe that the coordinated Mg^2+^ carrier, that is, [Mg(MeCN)_6_]^2+^ itself, could not migrate efficiently in the MOF because of the remaining electrostatic interaction with the framework, and also some of additional MeCN molecules strongly bound to the framework allow the formed Mg^2+^ carriers to migrate more freely (i.e., act as the conducting media). We also believe that the direct diffusion of the coordinated Mg^2+^ carriers assisted by some of the additional guest molecules is advantageous compared to the hopping of naked Mg^2+^, which requires reformation of the coordination bonds around Mg^2+^.

In conclusion, this is the first demonstration of super Mg^2+^ conductivity of around 10^–3^ S cm^–1^ in a porous MOF at ambient temperature. MIL-101⊃{Mg(TFSI)_2_}_1.6_ showed 1.9 × 10^–3^ S cm^–1^ at RT under MeCN vapor. We also determined the transport number of Mg^2+^ (*t*_Mg^2+^_ = 0.41), demonstrating that this is the first example of a solid-state crystalline super Mg^2+^ conductor with a practical conductivity of around 10^–3^ S cm^–1^ at RT (σ_Mg^2+^_ = 0.8 × 10^–3^ S cm^–1^). In situ FT-IR measurements, adsorption measurements, and partial pressure dependence of the ionic conductivity revealed that this super Mg^2+^ conduction is derived from the migration of coordinated Mg^2+^ species in the MOF pores and that some additional MeCN molecules adsorbed on the framework play a critical role in the efficient migration of the Mg^2+^ carriers. These results would greatly contribute to the development of the novel solid-state Mg^2+^ conductors operating at ambient temperatures.

## Supporting Information Available

XRPD data, Nyquist plots, ionic conductivity of various compounds, and adsorption isotherms (PDF)
