# Supplementary material for: Super Mg2+ Conductivity around 10–3 S cm–1 Observed in a Porous Metal–Organic Framework
Source: J Am Chem Soc. 2022 May 4;144(19):8669–75. doi: 10.1021/jacs.2c01612 (PMC9121370; doi:10.1021/jacs.2c01612)
Supplement: Supplementary file 1 — ja2c01612_si_001.pdf [file ja2c01612_si_001.pdf]

Supporting Information

Super Mg^{2+} Conductivity around 10^{-3} S cm^{-1} Observed in a Porous Metal–Organic Framework

Yuto Yoshida,[†] Teppei Yamada,[‡] Yuan Jing,[§] Takashi Toyao,[§] Ken-ichi Shimizu,[§]
Masaaki Sadakiyo*,[†]

[†]*Department of Applied Chemistry, Faculty of Science Division I, Tokyo University of Science, 1-3 Kagurazaka, Shinjuku-ku, Tokyo 162-8601, Japan.*

[‡]*Department of Chemistry, Graduate School of Science, The University of Tokyo, 7-3-1 Hongo, Bunkyo-ku, Tokyo 113-8654, Japan.*

[§]*Institute for Catalysis, Hokkaido University, N-21, W-10, Sapporo, Hokkaido 001-0021, Japan*

E-mail: sadakiyo@rs.tus.ac.jp

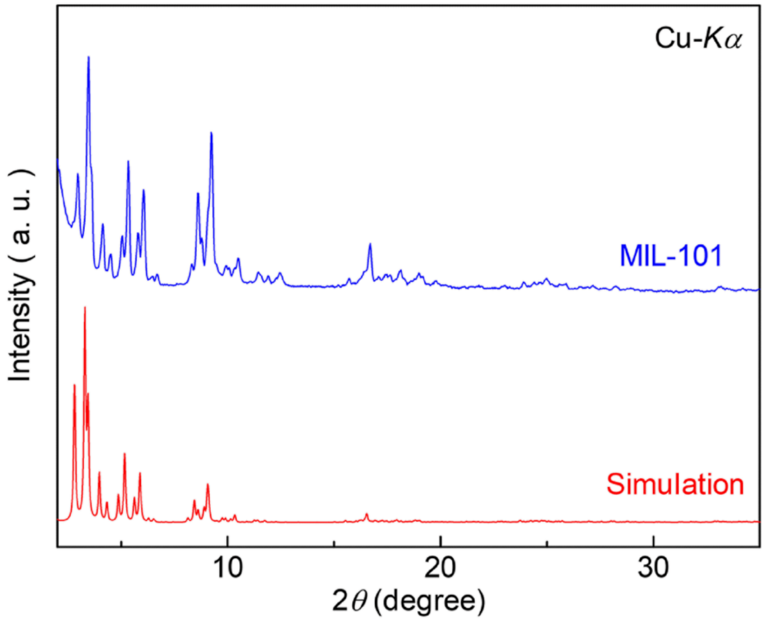

Figure S1. XRPD patterns of MIL-101. The red and blue colors correspond to the simulated pattern and the pattern of the prepared sample, respectively.

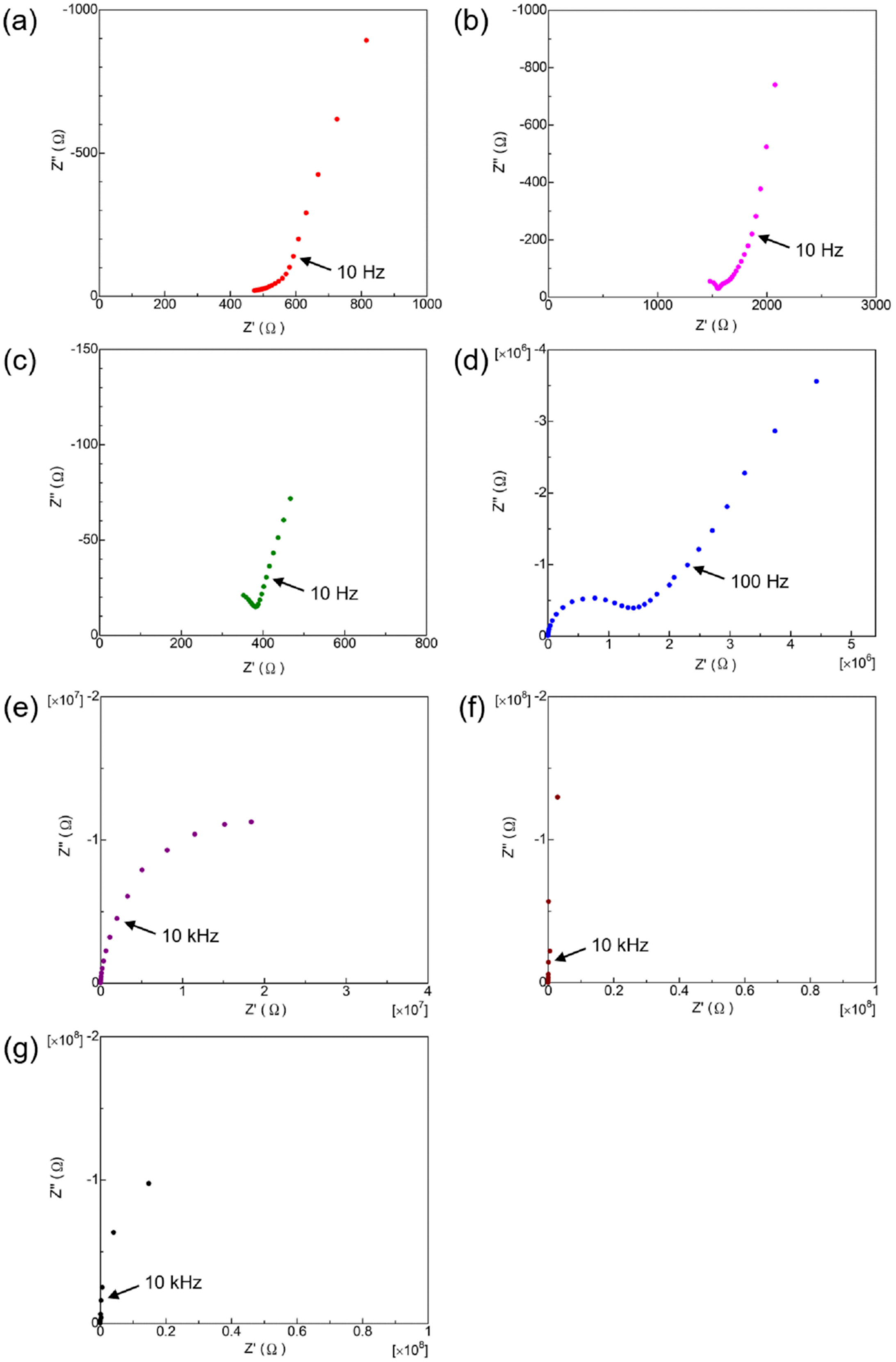

Figure S2. Nyquist plots of the x = 1.6 sample under (a) MeOH, (b) EtOH, (c) MeCN, (d) THF, (e) DEC, (f) PC, and (g) N_2; the example shown is at 25 °C.

Table S1. List of ionic conductivity of Mg^{2+}-included crystalline solids (LE = liquid electrolyte).

Material	Compound	Temp. (°C)	Conductivity (S cm^{-1})	E_a (eV)	t_{Mg2+}	Ref.
Tungstate	$MgHf(WO_4)_3$	600	2.5×10^{-4}	0.83	—	*J. Am. Ceram. Soc.* **2011**, *94*, 2285-2288.
Phosphate	$Mg_{0.5}Zr_2(PO_4)_3$	400	2.9×10^{-5}	0.82	—	*Solid State Ionics* **1987**, *23*, 125-129.
Phosphate	$Mg_{1.4}Zr_4P_6O_{24.4}$ + $0.4Zr_2O(PO_4)_2$	400	8×10^{-4}	1.40	—	*J. Mater. Chem.* **2000,** *10*, 1431-1435.
Phosphate	$Mg_{0.625}Si_{1.75}Al_{0.25}(PO_4)_3$	RT	1.54×10^{-5}	—	—	*J. Magnesium Alloys* **2017**, *5* , 439-447.
Borohydride	$Mg(BH_4)(NH_2)$	150	1×10^{-6}	1.31	—	*Chem. Commun.* **2013**, *50*, 1320-1322.
Borohydride	$Mg(en)_1(BH_4)_2$ (en = ethylenediamine)	70	6×10^{-5}	1.60	—	*Sci. Rep.* **2017**, *7*, 46189.
Borohydride	$Mg(BH_4)_2(NH_3BH_3)$	30	1.3×10^{-5}	1.47	—	*J. Phys. Chem. C* **2019**, *123*, 10756-10763.
Chalcogenide	$MgSc_2Se_4$	RT	1.0×10^{-4}	0.20	—	*Nat. Commun.* **2017**, *8*, 1759.
MOF + LE	Mg-MOF-74·$[\{Mg(TFSI)_2\}_{0.06}$ (+ triglyme)	RT	1.6×10^{-6}	0.13	—	*Energy Environ. Sci.* **2014**, *7*, 667-671.
MOF + LE	$Mg_2(C_{14}H_6O_6)$·$[\{Mg(TFSI)_2\}_{0.46}$·$Mg(OPhCF_3)_2\}_{0.21}]$ (+ triglyme)	RT	2.5×10^{-4}	0.13	—	*Energy Environ. Sci.* **2014**, *7*, 667-671.
MOF	$Cu_4(ttpm)_2$·$(CuCl_2)_{0.6}$·$(MgBr_2)_{0.7}$·21PC	RT	1.6×10^{-4}	0.24	—	*J. Am. Chem. Soc.* **2019**, *141*, 4422-4427.
MOF	Cu-azolate : $Mg_{0.5}[Cu_2Cl_2BrBTDD]$·8PC	RT	8.8×10^{-7}	0.37	—	*J. Am. Chem. Soc.* **2017**, *139*, 13260-13263.
MOF + LE	$Al_3O(OH)(BTC)_2$·$(Mg(ClO_4)_2)_{1.6}$·8.1PC	RT	1.0×10^{-3}	0.20	—	*ACS appl. Mater. Interfaces* **2020**, *12*, 43824-43832.
MOF	Mg-MOF-74·$\{Mg(TFSI)_2\}_{0.15}$ (under MeCN vapor)	RT	4.6×10^{-5}	0.32	0.47	*J. Phys. Chem. C* **2021**, *125*, 21124-21130.
MOF	Mg-MOF-74·$\{Mg(TFSI)_2\}_{0.15}$ (under MeOH vapor)	RT	2.6×10^{-4}	0.26	—	*J. Phys. Chem. C* **2021**, *125*, 21124-21130.
MOF	MIL-101·$\{Mg(TFSI)_2\}_{1.6}$ (under MeCN vapor)	RT	1.9×10^{-3}	0.18	0.41	This work

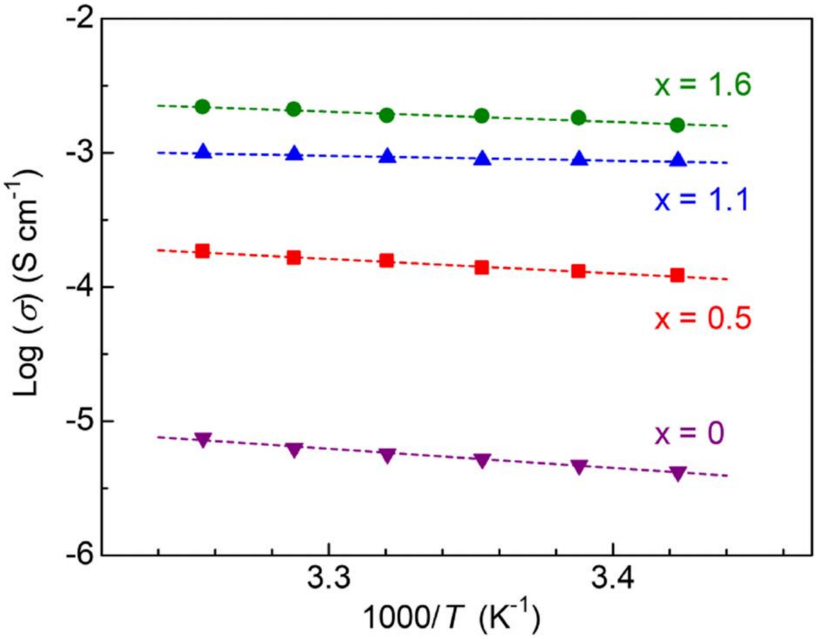

Figure S3. Temperature dependence of the ionic conductivity of the samples of x = 0–1.6 under MeCN vapor. Green circle, blue triangle, red square, and purple inversed triangle correspond to the ionic conductivities of x = 1.6, 1.1, 0.5, and 0, respectively.

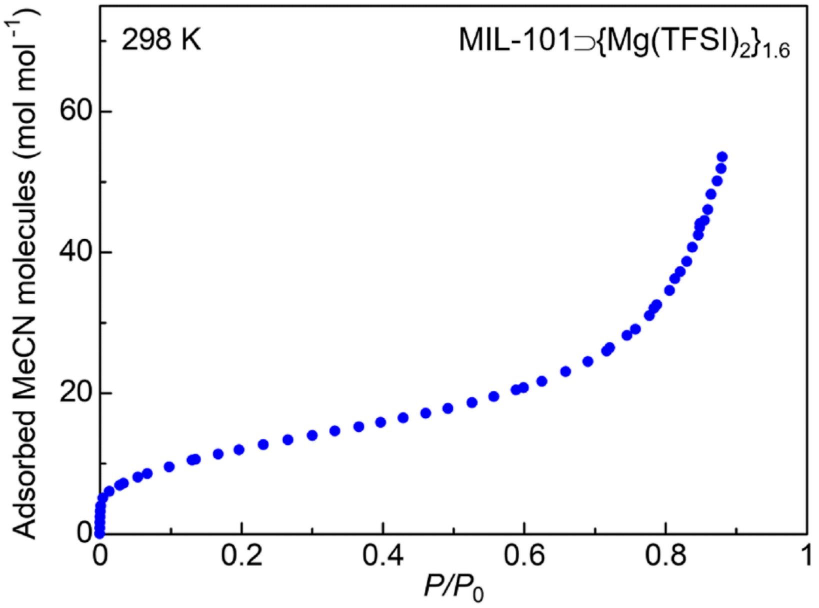

Figure S4. Adsorbed MeCN molecules in x = 1.6 at a various partial pressure of MeCN vapor at 298 K.

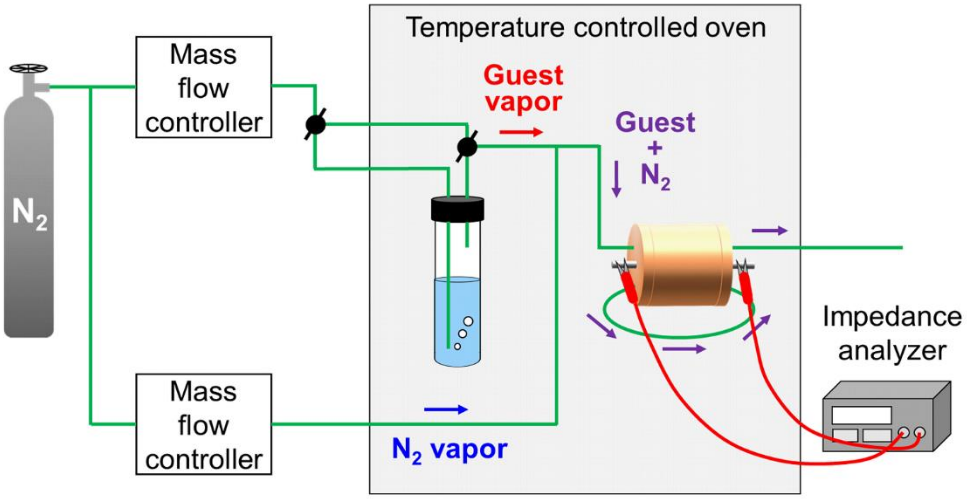

Figure S5. Schematic illustration of the experimental setup for evaluation of the partial pressure dependence of ionic conductivity.
